# Improved outcomes after groin hernia surgery in Sweden between 1992 and 2021: Swedish Hernia Register

**DOI:** 10.1007/s10029-025-03257-3

**Published:** 2025-01-09

**Authors:** Erik Axman, Henrik Holmberg, Erik Nilsson, Johanna Österberg, Ursula Dahlstrand, Agneta Montgomery, Pär Nordin, Hanna de la Croix

**Affiliations:** 1 Department of Pediatric Surgery, The Queen Silvia Children’s Hospital, Behandlingsvägen 7, 416 50 Gothenburg, Sweden; 2 Department of Surgery, Institute of Clinical Sciences, Sahlgrenska Academy, University of Gothenburg, Gothenburg, Sweden; 3 Department of Epidemiology and Global Health, Umeå University, Umeå, Sweden; 4 Department of Surgical and Perioperative Sciences, Umeå University, Umeå, Sweden; 5 Department of Surgery, Mora Hospital, Mora, Sweden; 6 Department of Clinical Sciences, Intervention and Technology (CLINTEC), Karolinska Institutet, Stockhom, Sweden; 7 Department of Surgery, Enköping Hospital, Enköping, Sweden; 8 Department of Surgery, Skåne University Hospital, Malmö, Sweden; 9 Department of Surgery, Sahlgrenska University Hospital/Östra Hospital, Gothenburg, Sweden

**Keywords:** Hernia, Nationwide register, Quality improvement, Recurrence, Laparo-endoscopic repair

## Abstract

**Purpose:**

Surgery for groin hernia is one of the most common operations in the world. Therefore, research concerning the outcomes of groin hernia surgery is extremely important both for the individual patient as well as for those providing the healthcare funding. The aim of this study is to evaluate the outcomes of hernia surgery in Sweden over a 30 year time period, from 1992 to 2021.

**Methods:**

All groin hernia repairs in the Swedish Hernia Register between 1992 to 2021 were analyzed with emphasis on the surgical method, reoperation rate for recurrence and date of surgery, specifically 1992–2001, 2002–2011 and 2012–2021. By using personal identification numbers, a cumulative reoperation rate has been deduced for males and females separately.

**Results:**

A total of 368,502 groin hernia operations identified in the Swedish Hernia Register between 1992 to 2021 were eligible for analysis. Since the register was begun, there have been significant changes in the choice of operative techniques, from suture repair in 1992 to open anterior mesh repair around the year 2000, until today, where an increasing proportion of hernias are repaired using laparo-endoscopic techniques. There has been a reduction in the reoperation rate for recurrence in both males and females, with the most pronounced improvement being seen in females. The laparo-endoscopic technique is associated with a reduced incidence of reoperation for recurrence in females.

**Conclusion:**

Groin hernia surgery in Sweden has undergone substantial changes over the past 30 years. Reoperation for recurrence has decreased significantly during recent years, especially in females.

## Introduction

The estimated lifetime risk for groin hernia is 27% for males and 3% for females. This results in approximately 20 million hernia operations each year, worldwide[18]. This magnitude represents significant healthcare costs, and even small improvements will have a large impact on public health. Swedish surgeons began to prospectively register groin hernia repairs in the Swedish Hernia Register (SHR) in 1992, making it possible to track important changes in practice, such as the introduction of mesh repair, laparo-endoscopic techniques, the use of local anesthetics, as well as the introduction of day surgery on a wider scale. There are other European and American registers, but with the exception of the Danish Hernia Database, the SHR is unique in its nationwide coverage, including all adult patients and operating units. This increases its external validity. A previously published validation of the register reveals a cover rate of 97%, and 98% correctly registered data [1]. The high external validity and a database comprising more than 370,000 repairs render the register a perfect tool for studying long-term outcomes, rare adverse events, and rare cohorts, such as hernias in females and femoral hernias.

The aim of this study was to analyze and describe the outcomes and characteristics of groin hernia surgery in Sweden over the past 30 years.

## Methods

### Registry and data description

The SHR was founded in 1992 with eight participating units. An initial audit of the register describes the rapid recruitment of participating units and the goal of improving hernia surgery across Sweden [16]. Since 2000, the register has had national coverage and currently more than 370,000 hernia repairs have been registered in individuals aged 15 years and older. Today, registration is performed by the surgeon online at the time of the procedure and includes preoperative information regarding age, body mass index, sex, American Society of Anesthesiologists (ASA) grade and operative data such as surgical technique, operative time, method of anesthesia and type of mesh used. Complications within 30 days are registered postoperatively by an independent coordinator. Using the Swedish personal identification number, a cumulative rate of reoperation for recurrence can be calculated regardless of where in Sweden the reoperations are performed and makes it possible to link the register to other registers [13]. The variables included in the register have changed somewhat over the years to ensure that relevant variables are included and to facilitate registration. Bilateral hernias are registered as two repairs, one on each side. In this article, TEP and TAPP repair is referred to as laparo-endoscopic technique while the Lichtenstein tension-free repair [12] is referred to as open anterior mesh repair. A combined repair refers to an open technique where both an anterior and posterior repair is used, such as the Prolene Hernia System and the patch and plug technique. The open preperitoneal repair includes several techniques including TransInguinal PrePeritoneal Tech-nique (TIPP), Nyhus and Stoppa. To guarantee quality and follow-up, one surgeon and a coordinator are appointed as registrars for all SHR registrations at each participating unit. The board of the SHR is composed of hernia surgeons, a registered nurse, and a medical secretary. A statistician, a platform administrator and a technical coordinator are co-opted members.

Annual validation of the data is carried out by professional validators who visit some 10% of the participating units, randomly selected. After validation, feedback is delivered to the registrar and the head of the validated surgical department.

This study followed the RECORD guidelines (Reporting of Studies Conducted using Observational Routinely-Collected Data) [4].

### Data collection and analysis

In this nationwide, register-based study, data from the SHR were retrieved from 1 January 1992 to 31 December 2021 and stratified by sex and date of surgery into three time periods (1992–2001, 2002–2011, and 2012–2021). Descriptive data are presented in tables and charts. A cumulative incidence of reoperation for recurrence is presented and stratified by date of surgery and operative technique. These are descriptive data from a national cohort. Therefore, no statistical analysis has been made to compare time periods or operative technique.

### Ethical considerations

Individuals being offered surgical repair for an inguinal or femoral hernia are informed of their participation in the SHR and can decline participation. They can also withdraw their consent later and have their data removed from the database. This study was approved by the Regional Ethics Committee in Gothenburg, Sweden, representing the Swedish Ethical Review Authority with reference numbers 417–17 and amendment 2022–04941-02.

## Results

**Fig. 1:**
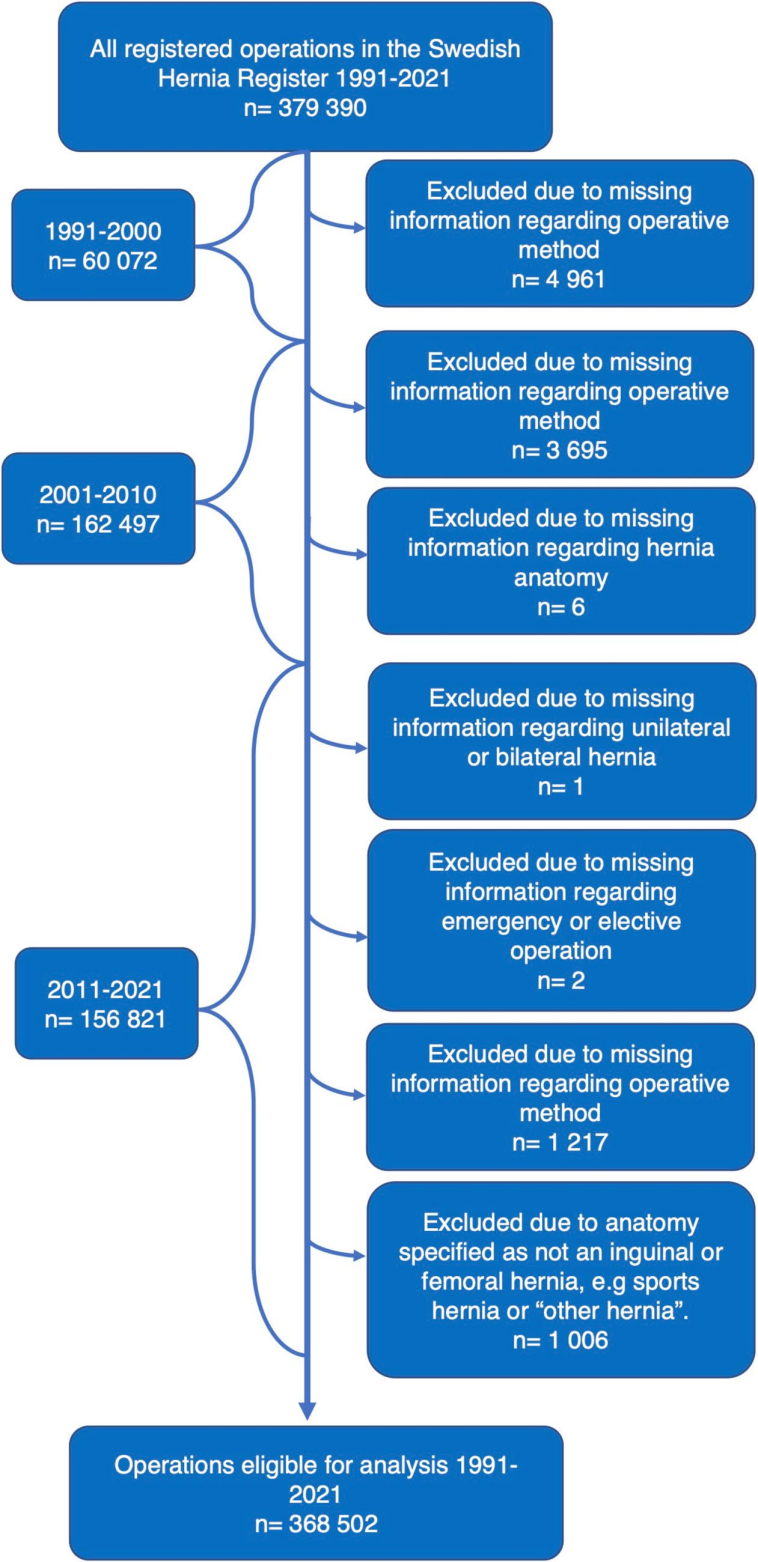
Flowchart describing inclusions and exclusions.

We identified 379,390 operations. In total, 10,888 operations were excluded due to missing data. After exclusions, 368,502 operations were eligible for analysis, as shown in Fig. 1.

Patient characteristics and operative data are shown in Table 1. The majority of operations, 338,199 (92%), were performed on males and 30,303 (8%) on females. There was no difference in age between the sexes with a mean age of 61 years. In females, the proportion of registered femoral hernias has increased over the years from 14% between 1992 and 2001 to 25% between 2012 and 2021. The proportion of registered medial hernias has simultaneously decreased from 23 to 17% over the same time periods. Most patients were ASA class I or II, with little difference between sex and surgery date. There was a substantial increase in the proportion of registered bilateral hernia repairs when comparing the period of 2012 to 2021 with the two earlier time periods. Bilateral hernia repairs were performed in 8.5% of all males from 1992 to 2001 and in 9.2% from 2002 to 2011. This should be compared to 14.8% bilateral repairs from 2012 to 2021. In females, 5.2% had bilateral repairs from 1992 to 2001, 6.1% from 2002 to 2011 and 16.1% in the period of 2012 to 2021.

**Table 1:** Preoperative, operative, and postoperative data on patients in the Swedish Hernia Register

	1992–2001		2002–2011		2012–2021	
	Males	Females	Males	Females	Males	Females
	(N = 51,789)	(N = 3322)	(N = 147,362)	(N = 11,434)	(N = 139,048)	(N = 15,547)
Age (years)						
Mean (SD)	58.9 (15.8)	58.8 (18.5)	59.9 (15.2)	59.9 (18.1)	61.7 (14.8)	61.7 (17.5)
Median [Min, Max]	60.0 [15.0, 98.0]	59.0 [15.0, 102]	61.0 [15.0, 102]	62.0 [15.0, 101]	64.0 [15.0, 102]	65.0 [15.0, 104]
ASA physical status						
ASA I-II	3 (0.0%)	0 (0%)	132,252 (89.7%)	10,363 (90.6%)	138,214 (99.4%)	15,395 (99.0%)
ASA ≥ III	0 (0%)	0 (0%)	573 (0.4%)	78 (0.7%)	834 (0.6%)	152 (1.0%)
Missing	51,786 (100.0%)	3322 (100%)	14,537 (9.9%)	993 (8.7%)	0 (0%)	0 (0%)
Anatomy						
Lateral	28,097 (54.3%)	1878 (56.5%)	78,845 (53.5%)	5901 (51.6%)	76,018 (54.7%)	8073 (51.9%)
Medial	18,612 (35.9%)	773 (23.3%)	53,391 (36.2%)	2683 (23.5%)	47,143 (33.9%)	2671 (17.2%)
Femoral	355 (0.7%)	466 (14.0%)	1510 (1.0%)	2205 (19.3%)	1936 (1.4%)	3895 (25.1%)
Combined (Lateral + Medial)	4015 (7.8%)	122 (3.7%)	12,849 (8.7%)	471 (4.1%)	12,032 (8.7%)	376 (2.4%)
Unspec	710 (1.4%)	83 (2.5%)	767 (0.5%)	174 (1.5%)	1919 (1.4%)	532 (3.4%)
Bilateral/Unilateral hernia						
Bilateral	4387 (8.5%)	172 (5.2%)	13,507 (9.2%)	692 (6.1%)	20,547 (14.8%)	2499 (16.1%)
Unilateral	47,402 (91.5%)	3150 (94.8%)	133,855 (90.8%)	10,742 (93.9%)	118,501 (85.2%)	13,048 (83.9%)
Reoperation for recurrence						
Yes	6692 (12.9%)	278 (8.4%)	14,209 (9.6%)	737 (6.4%)	12,914 (9.3%)	758 (4.9%)
No	45,097 (87.1%)	3044 (91.6%)	133,153 (90.4%)	10,697 (93.6%)	126,134 (90.7%)	14,789 (95.1%)
Repair technique						
Open anterior	23,585 (45.5%)	1007 (30.3%)	112,002 (76.0%)	6701 (58.6%)	93,216 (67.0%)	2345 (15.1%)
Combined*	7556 (14.6%)	542 (16.3%)	13,062 (8.9%)	1178 (10.3%)	3999 (2.9%)	404 (2.6%)
Laparo-endoscopic	6671 (12.9%)	277 (8.3%)	14,739 (10.0%)	1505 (13.2%)	38,092 (27.4%)	10,240 (65.9%)
Open non mesh	13,977 (27.0%)	1496 (45.0%)	7234 (4.9%)	1867 (16.3%)	887 (0.6%)	359 (2.3%)
Open pretroperitoneal	0 (0%)	0 (0%)	325 (0.2%)	183 (1.6%)	2854 (2.1%)	2199 (14.1%)
Anesthesia						
General	26,106 (50.4%)	1793 (54.0%)	101,258 (68.7%)	8402 (73.5%)	115,694 (83.2%)	14,534 (93.5%)
Local	6277 (12.1%)	345 (10.4%)	30,533 (20.7%)	1876 (16.4%)	19,006 (13.7%)	671 (4.3%)
Regional	19,405 (37.5%)	1184 (35.6%)	15,561 (10.6%)	1156 (10.1%)	4348 (3.1%)	342 (2.2%)
Missing	1 (0.0%)	0 (0%)	10 (0.0%)	0 (0%)	0 (0%)	0 (0%)
Emergency repair						
Yes	2328 (4.5%)	383 (11.5%)	6313 (4.3%)	1463 (12.8%)	5813 (4.2%)	1927 (12.4%)
No	49,461 (95.5%)	2939 (88.5%)	141,049 (95.7%)	9971 (87.2%)	133,235 (95.8%)	13,620 (87.6%)

*ASA* *American Society of Anesthesiologists SD Standard deviation*

^*^combined: Anterior and posterior approach, plug and patch, PHS-system.

**Fig. 2:**
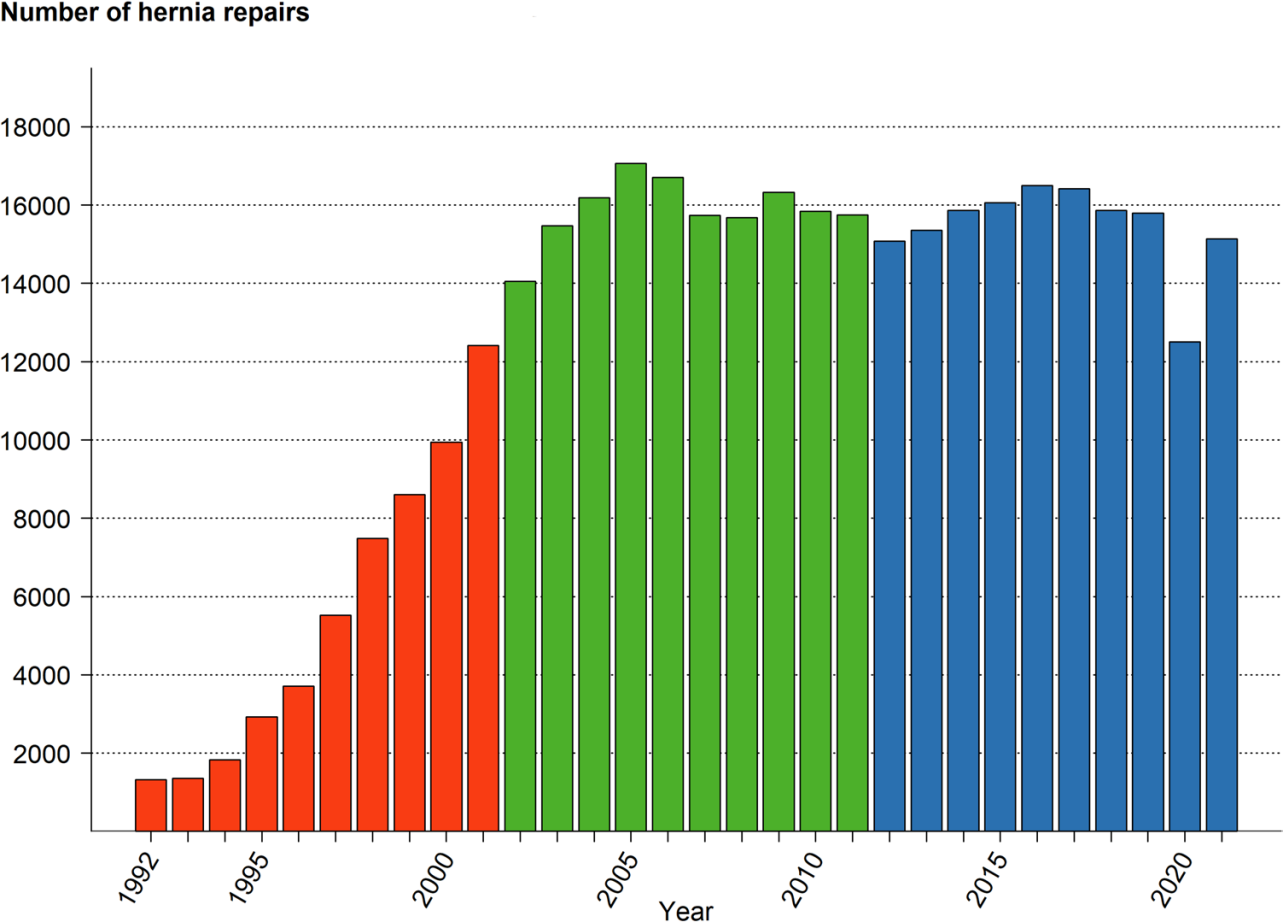
Number of hernia repairs in the Swedish Hernia Register 1992–2021.

The annual number of registered operations in the SHR increased from the start of the register until 2005. Since then, the number of operations registered each year has varied little, except for the pandemic in 2020, as seen in Fig. 2.

**Fig. 3:**
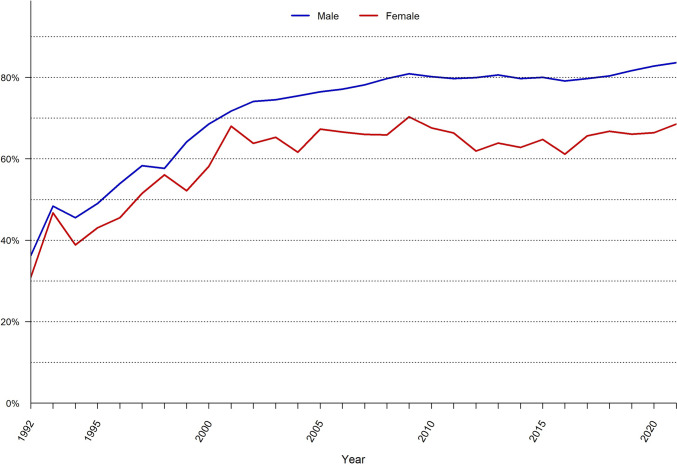
Proportion of outpatient surgery in the Swedish Hernia Register 1992–2021.

Over time, general anesthesia and local anesthetics have increased as the use of regional anesthetics has decreased. Over the entire time period, females had a higher rate of emergency repair, namely 15% versus 4.5% in males. Since the year 2000, most operations have been performed as day surgeries, as illustrated in Fig. 3.

## Hernia repair techniques

**Fig. 4:**
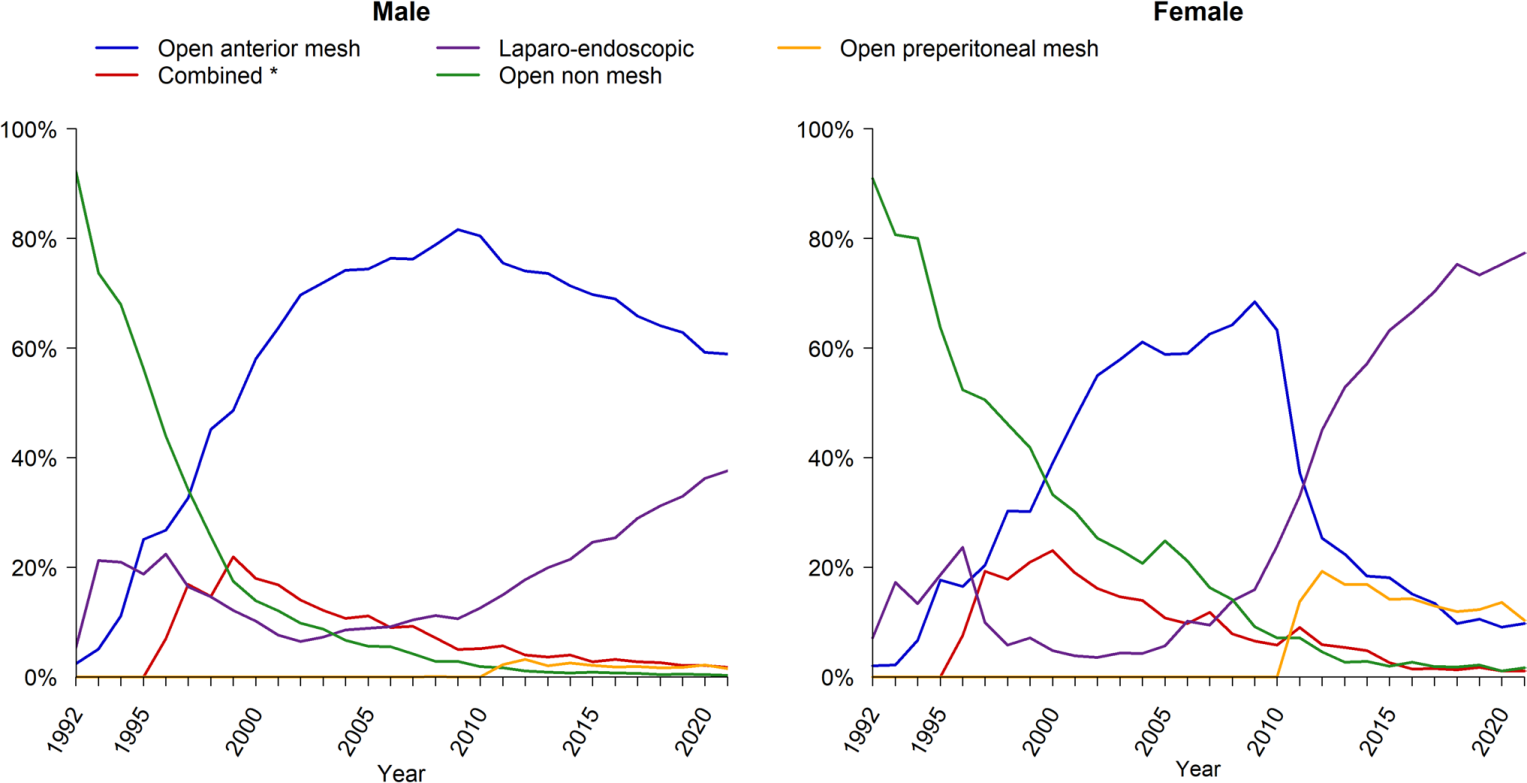
Groin hernia repair techniques in the Swedish Hernia Register in males and females 1992–2021

Repair methods have varied greatly over time and differ in males and females, as illustrated in Fig. 4. For males, sutured hernia repair was largely replaced by the Lichtenstein repair, referred to as open anterior mesh repair, between 1992 and 2001. From 2010 to 2021, open anterior mesh repair was still the predominant repair method but this decreased over time as the use of the transabdominal preperitoneal patch technique (TAPP) and total extraperitoneal technique (TEP) increased. A similar development was seen in females from 1992 to 2001. Open anterior mesh repair in females decreased abruptly from 2008 onwards at the same time as TEP and TAPP increased in frequency and by 2011 laparo-endoscopic repairs were the most common type of repair in females. Almost all laparo-endoscopic repairs in Sweden are performed using the TEP technique.

## Reoperations due to recurrence

**Fig. 5:**
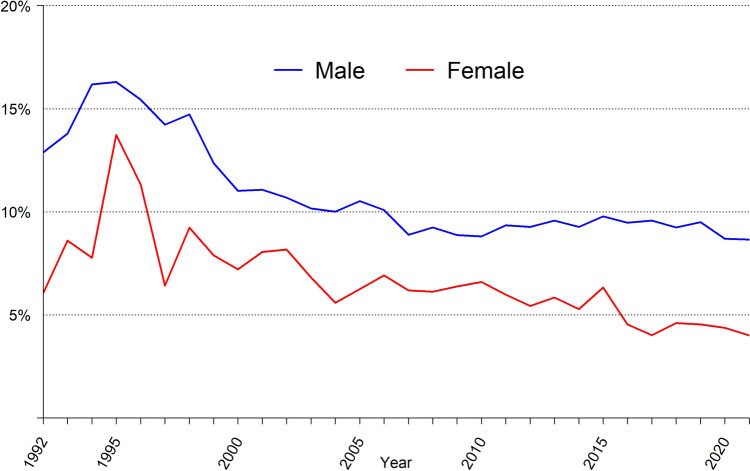
Proportion of operations performed due to recurrence in the Swedish Hernia Register 1992–2021.

The proportion of recurrent hernia operations decreases over time in both males and females, as shown in Fig. 5. After 2016, less than 5% of all operations performed on females were because of recurrences. In males, less than 10% of all operations were recurrent hernias after 2006. This should be compared to 17% when the register started[16].

**Fig. 6:**
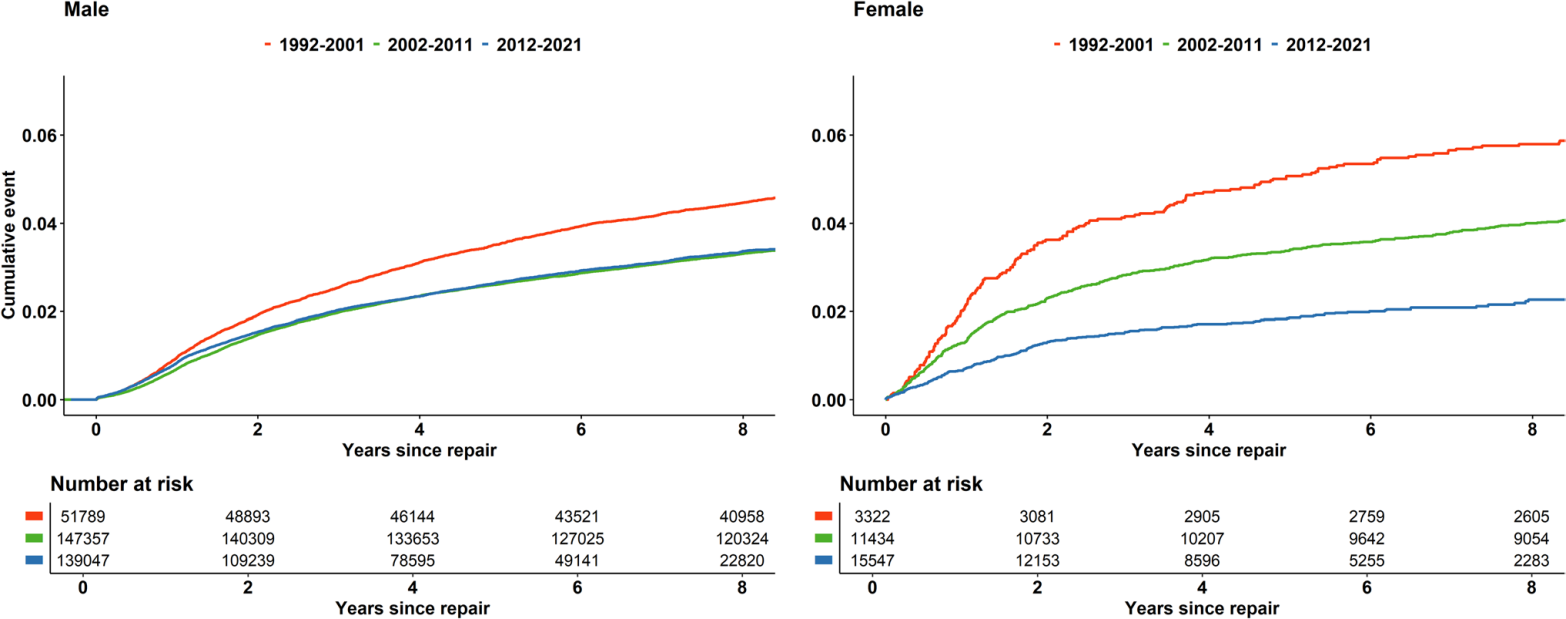
Reoperation for recurrence after groin hernia repair in the Swedish Hernia register for males and females 1992–2021.

The rate of reoperation for recurrence was similar for males when comparing the periods 2002–2011 and 2012–2021, while the earliest period, from 1992 to 2001, presented a higher rate of reoperation due to recurrence. In females, each time period presented a clear reduction in the rate of reoperation for recurrence, as compared to the previous period (Fig. 6).

**Fig. 7:**
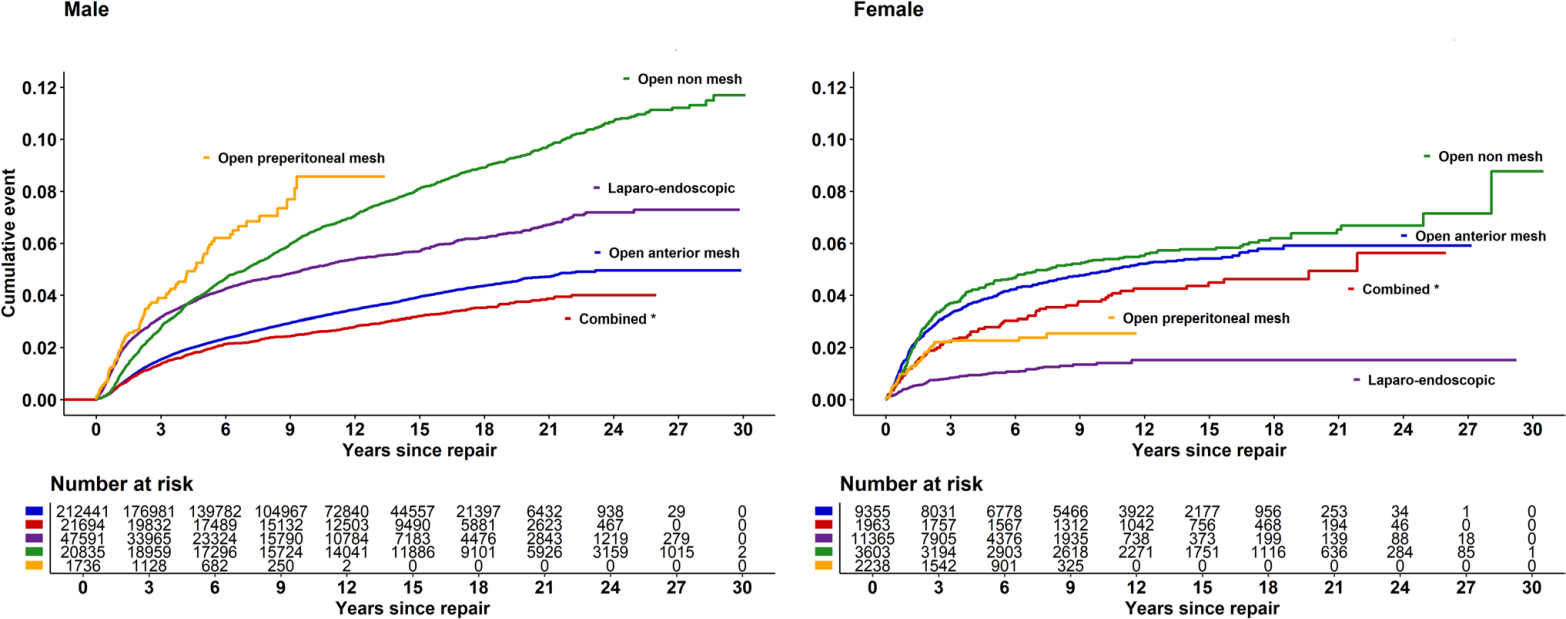
Reoperation for recurrence after primary groin hernia repair, separated by repair technique in the Swedish Hernia register for males and females 1992–2021.

The cumulative rate of reoperation for recurrence for primary operations, stratified by gender and operative technique, is shown in Fig. 7. For males a combined repair or an open anterior mesh repair represents the lowest rates of reoperation for recurrence. An open preperitoneal or an open non-mesh repair represents the highest rates of reoperations for recurrence. The laparo-endoscopic techniques, TEP and TAPP, show fewer reoperations for recurrence than open non-mesh repair and open preperitoneal repair. In females, the laparo-endoscopic techniques are superior to all other methods of repairs, as is clearly illustrated in Fig. 7.

## Discussion

Great changes and significant improvements have been made in groin hernia surgery over recent decades and, for the first time, this is illustrated by using a quality register with national coverage and 30 years of follow-up. Major changes can be seen in the choice of operative method, where non-mesh repairs have been almost completely replaced by mesh repairs. In women, the increased use of endoscopic techniques has coincided with an increase in registration of femoral hernias, a decrease in registration of medial hernias and a clear reduction in the rate of reoperation because of recurrence. In men, the rate of reoperation was reduced significantly over the register’s first 10 years but has stayed surprisingly constant throughout the past 20 years.

Only the Danish Hernia Register with full national coverage has found similar improvements in groin hernia surgery on a national level. Between 1992 and 2001, the proportion of recurrences versus primary operations decreased from 17 to 14% in Denmark, comparable to the 13.5% seen in our study [3].

An increase in the rate of operations for bilateral hernias can be seen. Current European guidelines recommend a laparo-endoscopic approach to repair bilateral hernias and the increased use of laparo-endoscopic repair in Sweden might explain the increase of bilateral repairs over the years[22]. One other possible explanation could be the increased use of laparo-endoscopic techniques, which allow an improved intraoperative assessment and visualization of bilateral hernias.

The introduction of the Lichtenstein tension-free repair[12] has been widely adopted by hernia surgeons worldwide including Swedish surgeons, and is referred to as open anterior mesh repair in this article. Recommendations issued by Sweden’s National Board of Health and Welfare in 2011 stated that female patients and bilateral hernias for both males and females should be offered a posterior approach with TEP or TAPP. This, together with similar recommendations from the Danish Hernia Database in 2011[20], is likely to have been the cause of the marked increase in the use of a laparo-endoscopic techniques in females. Since these recommendations were made, even more evidence has emerged supporting the use of laparo-endoscopic techniques in females[21]. During a time period of ten years, from 2011 to 2021, the proportion of laparoscopic hernia repair in females increased from 40% to 80%. The learning curve and the proportion of emergency repairs are possible factors reducing the rate of transition to laparoscopic repair for females on a national level. The increase in laparo-endoscopic techniques is less pronounced in men. The use of laparo-endoscopic techniques in men is much lower in Sweden than in Denmark, 35% vs 65% [6, 23]. A previous study from SHR showed TEP to be associated with a decreased risk of reoperation because of recurrence in women, but not in men, for whom it significantly increased the risk of reoperation after TEP. As seen in Fig. 5, the increase of laparo-endoscopic techniques in males coincides with an end to improvements regarding risk of reoperation. The fact that most males are offered an open operation might be explained by the presentation of evidence that suggests its benefits.

When comparing the three different periods, the reduction in reoperations because of recurrence seen in females is remarkable, and there could be several reasons for this. Before hernia registers were initiated, few studies included females and the choice of treatment was based on data from studies performed on males. Using large register studies based upon national registers, it has become possible to study large cohorts of females with groin hernias, and this has changed clinical practice radically[2]. The initial improvement seen in our data could possibly be explained by the introduction of the mesh repair. The continued improvement between 2002–2011 and 2012–2021 was caused by the widespread implementation of laparo-endoscopic techniques in females as shown in earlier studies[17] and illustrated in Fig. 4, where the changes in the repair technique coincide with the reduction of reoperations. This change of practice was based upon a hypothesis that many of the reoperations after inguinal hernia repairs in females were, in fact, missed femoral hernias[9]. Our study confirms this with a significant increase in the proportion of registered femoral hernias in females, from 14% 1992–2001 to 25% at the end of the study period. These findings represent a significant increase in the quality of treatment for groin hernias in females.

This study shows that the choice of operative technique has a significant impact on the incidence of reoperations for recurrence. It is also evident that a long follow-up time is needed to evaluate the quality of the operative technique in terms of reoperation rates. For females, a laparo-endoscopic technique results in the lowest rates of reoperation for recurrence. The fact that it is now possible to identify a femoral hernia, and that TEP and TAPP surgery in females is performed mainly by experienced surgeons, might be an explanation for these results.

For males, the higher rates of reoperations for recurrence seen with TEP and TAPP as compared to an open anterior mesh repair might be explained by a longer learning curve. Laparo-endoscopic techniques have been adopted by many surgeons but are not necessarily concentrated in high volume centers, and this may be required to improve the results. Additionally, TEP and TAPP may include a more challenging dissection in males due to the spermatic cord and testicular vessels, as well as the presence of larger hernias such as scrotal hernias. This might contribute to the inferior results with TEP and TAPP in males compared to females.

Very few studies exist that report the long-term reoperation rates for a larger cohort stratified by operative technique. A study from the Danish Hernia Database showed significantly lower rates of reoperation for recurrence for Lichtenstein repair versus sutured repair when examining the 5–8 year results from 1998 to 2005[5]. Reoperation for recurrence rates are similar to those in this study. Interestingly, the same pattern appears with a sutured repair showing a continuous increase in recurrence rates compared to the Lichtenstein repair where the majority of recurrences are reoperated within the first 5 to 8 years.

Day surgery in hernia patients is considered safe and cost efficient[11, 14, 19] and our results show a significant increase over time. Over the past 10 years, approximately 80% of all operations have been performed as day surgeries. For females, the proportion of day surgery is lower, around 60% to 70%. This might be explained by the higher proportion of emergency surgery in females. Day surgery varies between countries and there are reports of increasing rates from, for example, the Netherlands, from 39% in 2001 compared to 54% in 2005[7]. The reported rates for day surgery may suggest that most patients in any country can be offered day surgery without severely impacting results.

One possible explanation for some of the improvements in the reduced rate of reoperations seen in this study might lie in the registration itself. The so-called “Hawthorne effect” is created when people adjust aspects of their behavior because of their awareness of being observed. This was shown in one of the first publications based on the register. The risk of reoperation for the initial eight hospitals was compared to that of the next eight hospitals that joined the register in 1995. The hospitals that joined the SHR in 1995 showed similar improvements as the initial eight hospitals during their first three years of registering[15].

Our study has several strengths, including the combination of a uniquely large number of patients, national coverage and the long follow-up time. Using the Swedish personal identification number makes it possible to detect most reoperations for recurrence, thereby allowing us to describe the effectiveness of the surgical treatment on a nationwide scale. Our data represent the results of hernia surgeries in Sweden over a 30-year period. Another advantage is the relative consistency in data collection, with only minor changes in the clinical variables that are included. Therefore, the study findings are easy to evaluate over time.

Besides the Swedish Hernia Register, there are several hernia registers around the world, all with different properties and strengths which are well described in the CORE project[10]. There are similarities and differences between the registers regarding, for example, funding and the cover rate relative to the country’s population. The register most similar to the SHR is the Danish Hernia Database which is also publicly funded, uses a national personal identification number and has a cover rate of approximately 90% of the country[8]. The SHR is funded by the government without the involvement and potential bias of medical companies.

A weakness of this study is that the data presented are only as valid as the quality of the data in the register. Although the data quality and coverage rate for the SHR have been evaluated[1], there is an inborn risk of errors when multiple individuals include data, as is the case with registry data. The study design makes it possible to describe the results, but it will not provide evidence regarding the cause of the observed changes. Furthermore, our study results are valid for Sweden but might not be valid in other countries with different settings. The SHR is also limited in that recurrences per se are not detected but only reoperations for recurrence.

In the future, the Swedish Hernia Register aims at further improving hernia surgery by the introduction of registry-based randomized clinical trials. Moreover, to improve the previous patient-related outcome measures, a new instrument called HERO is being developed, combing both pre- and postoperative patient-related outcome measures.

This study presents a unique overview of the changes in groin hernia surgery in Sweden over the past 30 years. Considerable changes have been made in the operative techniques, particularly the use of mesh repair and the introduction of laparo-endoscopic techniques. A consistent reduction in the rate of reoperation for recurrence has been seen, with females showing the most dramatic improvements. This study highlights the benefits of national registers for evaluating results over time and for a large, unselected population.
